# Educators’ Interprofessional Collaborative Relationships: Helping Pharmacy Students Learn to Work with Other Professions

**DOI:** 10.3390/pharmacy4020017

**Published:** 2016-03-30

**Authors:** Anne Croker, Tony Smith, Karin Fisher, Sonja Littlejohns

**Affiliations:** 1 Tamworth Education Centre, Department of Rural Health, University of Newcastle, 114-148 Johnston Street, Tamworth, NSW 2340, Australia; Karin.Fisher@newcastle.edu.au (K.F.); Sonja.Littlejohns@newcastle.edu.au (S.L.); 2 Manning Education Centre, Department of Rural Health, University of Newcastle, 69A High Street, Taree, NSW 2430, Australia; Tony.Smith@newcastle.edu.au

**Keywords:** co-located settings, educators, interprofessional education, interprofessional learning, pharmacy, relationships, workplace learning, work integrated learning

## Abstract

Similar to other professions, pharmacy educators use workplace learning opportunities to prepare students for collaborative practice. Thus, collaborative relationships between educators of different professions are important for planning, implementing and evaluating interprofessional learning strategies and role modelling interprofessional collaboration within and across university and workplace settings. However, there is a paucity of research exploring educators’ interprofessional relationships. Using *collaborative dialogical inquiry* we explored the nature of educators’ interprofessional relationships in a co-located setting. Data from interprofessional focus groups and semi-structured interviews were interpreted to identify themes that transcended the participants’ professional affiliations. Educators’ interprofessional collaborative relationships involved the development and interweaving of five interpersonal behaviours: *being inclusive of other professions; developing interpersonal connections with colleagues from other professions; bringing a sense of own profession in relation to other professions; giving and receiving respect to other professions;* and *being learner-centred for students’ collaborative practice*. Pharmacy educators, like other educators, need to ensure that interprofessional relationships are founded on positive experiences rather than vested in professional interests.

## 1. Introduction

Similar to many professions in healthcare, pharmacy needs to work with other professions in order to improve interprofessional collaborative practice [1] and prepare students for such practice [2]. Interprofessional collaborative practice is integral to reducing the risk of clinical error [3] as well as dealing with changing drivers of healthcare, which include ensuring resource efficiency, knowledge expansion, and valuing practitioners and consumers in healthcare [4]. With their roles in the frontline of primary healthcare, pharmacists are well placed to work with other professions and address these challenges; for example, by reducing inappropriate polypharmacy through multidisciplinary case conferencing [5], and pharmacist-led medication reviews [6,7]. Information availability and patient-centred views of healthcare are at the forefront of pharmacy’s aim to ensure user-friendly, evidence-based information about medications that can facilitate patients’ empowerment and participation in decision-making [8]. Collaboration with pharmacists in healthcare teams is a positive step towards improved therapeutic safety and humanistic healthcare outcomes [9]. However collaboration in healthcare is complex [10]. Common challenges faced by health professionals, including pharmacists, are conflicting professional agendas, communication difficulties and insufficient resources [11]. Barriers to integrating pharmacists into primary care teams include unclear role definition, boundary encroachment, poor communication, lack of time, lack of infrastructure and payment issues [12,13,14]. Correspondingly, ongoing funding, adequate space, clear role definition, pre-existing relationships and characteristics, as well as the attitudes of individual practitioners have been identified as factors that enable collaboration [15].

Ideally, interprofessional education (IPE) strategies, as part of work integrated learning (WIL), create opportunities for students to experience interprofessional collaboration and learn to constructively address challenges. IPE is “*when two or more professions learn with, from and about each other to improve collaboration and the quality of care*” [16]. IPE initiatives involving pharmacy students on WIL placements include formal strategies, such as student-led clinics [17] and interprofessional learning sessions integrated into rural placements [18,19], as well as less formal strategies, such as workplace role modelling [20]. The American College of Clinical Pharmacy highlighted the importance of workplace role modelling: “*clinical faculty and other practitioners with extensive experience in interprofessional practice serve in critical roles as mentors and as role models*” [20] (p. 149e). Supporting the value of IPE is the following feedback from pharmacy students after a rural interprofessional WIL experience: “*I now understand how important it is for healthcare workers to work in collaboration for the health of the patient*” and “*I now have an understanding of the roles of the different professions*” [21].

Collaborative relationships between educators of different professions are also, arguably, of paramount importance in planning, implementing and evaluating IPE initiatives and for role-modelling positive interprofessional interactions. Linqvist and Reeves [22] identified the importance of previous experiences with collaboration in health care teams for informing facilitators’ work with students. Freeth and Reeves [23] recognised the influence of educators’ views on collaboration for the design and delivery of educational experiences and cautioned about assumptions and stereotypes being unconsciously projected. However, despite the insights and cautions of these authors, educators’ collaborative relationships are rarely the explicit focus of IPE literature [24]; rather they tend be an implicit component, as was our experience in previous research.

In our previous research we identified the importance of interprofessional rapport between students’ as they learned to work with other professions [19]. Interestingly, interprofessional rapport also appeared relevant to educators’ collaboration, however clarifying this relevance was beyond the scope of that particular study. Accordingly the research was extended to explore the nature (that is, inherent features, characteristics or qualities) of interprofessional collaborative relationships between educators, including pharmacy academics, who work across the university and clinical sectors and are involved in an IPE program that has been successfully delivered for more than a decade. We wanted to explicitly explore the relevance for educators of the students’ interprofessional rapport building capabilities identified in our initial study [19].

The setting of this research is the University of Newcastle, Department of Rural Health (UONDRH), which is part of the Australian Government’s Rural Health Multidisciplinary Training program [25]. The UONDRH is located in a rural community with a population of around 50,000. Students attending the UONDRH are enrolled in either diagnostic radiography, medicine, nursing, nutrition and dietetics, occupational therapy, pharmacy, physiotherapy or speech pathology. Placements vary from a week to a full academic year, depending on program requirements. Remote from the main university campus (approximately 280 km away), UONDRH students live, socialise and undertake WIL and community engagement activities in multi-professional, as well as profession-specific groups. Onsite accommodation is available for 57 students who are predominantly undertaking full year or semester long placements, with off-site accommodation available for a further 19 short-term students.

The UONDRH employs academic educators in all the above professions, as well as administrative staff. The educators deliver interprofessional learning (IPL) modules on a regular basis and often teach into health professional courses other than in their own discipline. They share office and teaching space and work closely with their clinical colleagues to support students’ workplace learning in a variety of public, private and non-government healthcare settings. The close links with clinical colleagues is enabled by educators having part time academic appointments with the UONDRH and part time clinical appointments with healthcare services. Pharmacy educators are an integral part of the interprofessional team in planning and delivering IPL modules, as well as teaching prescribing tutorials to senior medical students and medication management to students in other disciplines.

This qualitative study addresses the following research question: “What is the nature of educators’ interprofessional collaborative relationships in a co-located setting, as they help students learn to work with other professions?” Underpinning the exploration of educators’ interprofessional collaborative relationships is the importance of ensuring that educators can say “do as we do AND as we say” in relation to IPE within WIL. Findings will inform ongoing collaboration as the educators of different professions continue to work together to facilitate IPE initiatives and role model interprofessional relationships.

## 2. Method

Collaborative dialogical inquiry [26] was used to enable the research to be undertaken “with” the educators rather than “about” them. As co-researchers, educators had ownership of and responsibility for research decisions, actions and outcomes through a dialogical process, enabling ideas and insights to be identified, challenged and reshaped. The underpinning assumption behind the choice of this research method was that co-produced knowledge developed by including educators who were participants in the initial study as co-researchers would enhance the relevance of research outcomes for changes to their IPE and WIL practices.

Twenty one educators from UONDRH participated in the collaborative dialogical inquiry, 17 of whom have roles in both academic and healthcare settings, while four have roles only in the academic setting. The team of co-researchers comprised 19 females and 2 males. All disciplines in the UONDRH program were represented as follows: diagnostic radiography (*n* = 2), medicine (*n* = 4), nursing (*n* = 4), nutrition and dietetics (*n* = 3), occupational therapy (*n* = 2), pharmacy (*n* = 2), physiotherapy (*n* = 3) or speech pathology (*n* = 1).

**Figure 1:**
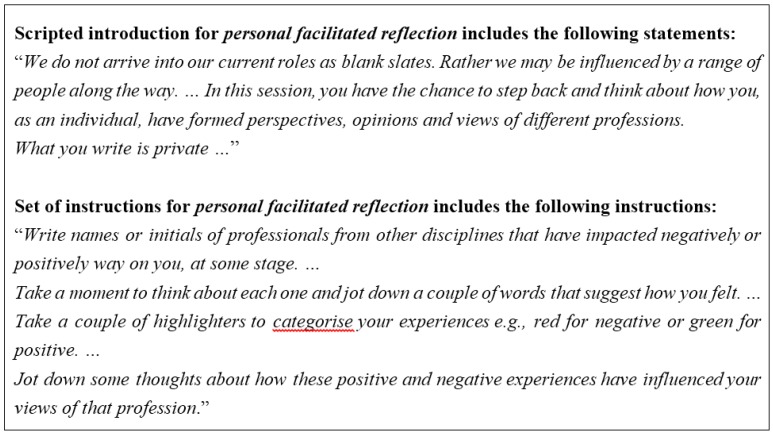
Examples statements and instructions from educators’ *personal facilitated reflection*.

Collaborative dialogical inquiry has four iterative stages [27]: (i) involvement of the research group; (ii) cohesion of the research group; (iii) immersion in issues for development of practice; and (iv) consolidation of changed practice (this final stage is still in progress). Dialogue between the co-researchers within and across these four phases involved telling, listening, sharing and questioning, as part of ongoing conversations, critical debate and group reflection. Trust developed in stage (ii) of the collaborative dialogical inquiry was important for the openness of this dialogue. Some of these conversations and group reflections were formally arranged focus groups, while others were more informal opportunistic discussions that reflected the interactive nature of the research group. In preparation for some of the focus groups co-researchers participated in a “personal facilitated reflection” (see Figure 1).

Focus groups, run by principal researcher (AC), became data sources for formal interpretation by AC. AC has no educational role or staff responsibilities in the UONDRH that would impede participants’ and co-researchers’ honesty. Additional data sources were 24 photo-elicitation interviews and three interprofessional educator focus groups undertaken in the initial study (these were re-analysed to explicitly explore educators’ interprofessional collaborative relationships), as well as AC’s reflective journal from both studies. An overview of the interview and focus group data sources, according to profession, is provided in Table 1. Questions posed to educators in focus groups included: have you ever questioned your perspectives on other professions; to what extent did you learn about other professions as part of the IPL modules; have your previous experiences with other professions influenced your current involvement with interprofessional rapport building opportunities; I would like to explain some of the emerging themes; what are your thoughts about this theme; does this theme resonate with you; what would you like to challenge about it; and does it raise any issues for you?

Interviews and focus groups were professionally transcribed. Following transcription, AC listened to the audio recording to check for accuracy and engage with the complete transcription. AC’s interpretation of data sources (similar to the earlier study see [19]) was informed by philosophical hermeneutics [26] and involved AC posing questions to the text (dialogue of questions and answers), moving iteratively between new insights obtained from a particular section of texts to her whole understanding (moving between parts to the whole) in order that her initial understandings were fused with the meanings provided by the texts (fusion of horizons). Questions posed to the texts during the iterative cycles of interpretation included: when, how and why do educators relate to each other; what qualities do they need to relate to each other; and what happens over time? Through this ongoing interpretation the initial descriptive codes were refined into conceptual codes, iteratively discussed and critiqued by co-researchers in conversations and focus groups then organised into findings. Due to the part-time employment of many researchers, care was required in scheduling of focus groups and in undertaking informal discussions to ensure all researchers had a voice. The iterative nature of these conversations and focus groups were important for ensuring the robustness of the interpreted themes.

Ethics clearance for both studies was obtained by the University of Newcastle Human Research Ethics Committee and the Hunter New England Human Research Ethics Committee. All data are securely stored and are reported anonymously.

## 3. Findings

Educators’ interprofessional relationships, important for helping students learn to work with other professions, involved the development and interweaving of five interpersonal behaviours, each building on the other: *being inclusive of other professions; developing interpersonal connections with colleagues from other professions; bringing a sense of own profession in relation to other professions; giving and receiving respect to other professions;* and *being learner-centred for students’ collaborative practice*. Despite the sequential representation of these behaviours, educators did not necessarily begin at similar points or progress in the same manner, nor was progression necessarily linear.

Each of the five interpersonal behaviours identified in this research are described below, with quotations from participants. These interpersonal behaviours extend beyond professional affiliations and educational roles (both in academic and healthcare settings), often relating to previous experiences with practitioners from other professions. Due to the non-profession-specific nature of the quotes, professional affiliations are not specified for the quotations.

### 3.1. Being Inclusive of Other Professions

*Being inclusive of other professions* was not necessarily a familiar practice for all educators when they began their involvement with interprofessional learning:

“Well the main profession that I work with is [particular profession] so [they are] my first and probably my major contact. I don’t have a lot to do with other [professions] in my day to day duties. … I know going way back when I was at uni, we didn’t have a lot of interaction with the other disciplines, socially or in combined lectures or whatever.”

(Educator N28)

Without previous experiences with interprofessional learning, their profession-centric focus in clinical and educational settings may not be obvious to them.

“I think before I came here I was in one of those silos … I think I wasn’t aware that I was in it.”

(Educator G9)

Through positive experiences educators’ attitudes could change and become more inclusive of other professions.

“My perspective has changed on one particular profession. … There’s more depth to what they actually do.”

(Educator G9)

“I think working with the other health professions (educators) has made me teach the importance of not being so tunnelled into your own profession.”

(Educator H39)

However being inclusive is not necessarily easy or straightforward when there have been negative experiences with particular professions in the past. Discipline territory issues can be the source such difficulties.

“When I see people behaving as if they own a scope of practice or a skill … I find that extremely irritating.”

(Educator H8)

Being upfront with such issues and the implications for patient care was one way of addressing such issues:

“Some things (about discipline territories) you can accept and some things you can’t. (But) you come at it from the patient perspective, what’s better for the patient … you need to be able to question another profession.”

(Educator O18)

### 3.2. Developing Interpersonal Connections with Colleagues from Other Professions

*Developing interpersonal connections with colleagues from other professions* was an important aspect of educators learning to work together. Knowing educators personally can be helpful for breaking down professional barriers.

“If you have preconceived ideas (about particular professions) that are negative, I’ve found that … (knowing an individual from that profession through working here) has broken down some barriers that I’ve internally had.”

(Educator M15)

Blurring boundaries between work and social situations can also be helpful for understanding other professions.

“I think it’s learning more about the person in order to make their profession more accessible. I think if you have a friend or know of someone socially, and they’re in a particular profession you’re going to probably know a little bit more about that profession, particularly if you’re quite friendly with that person.”

(Educator G9)

These interpersonal connections enabled boundaries to be blurred between university and clinical workplaces, and between workplace and social situations.

“I suppose as working for [UONDRH] there’s probably an expectation to come to these social things. You don’t have to but I enjoy them.”

(Educator C29)

However, as part of knowing each other well, educators also needed to be aware of inadvertently misrepresenting interprofessional relationships to students.

“Because we all know each other here and we’re friendly toward each other we might sling off as a joke [about each other’s profession] but the students may not realise we’re joking.”

(Educator N4)

### 3.3. Bringing a Sense of Own Profession in Relation to Other Professions

Educators appreciated being able to represent the scope and contributions of their particular professions, opportunities often offered in formal interprofessional learning situations.

“[In our interprofessional learning modules] you’re your own person to shine within your area and each person has that opportunity to be that person and promote their own profession which is a fantastic opportunity really for people to recognise the whole patient pathway and not just the bit that they do.”

(Educator O18)

However, in isolation this sense of own profession was insufficient, it needed to be balanced by taking care to ensure that the contributions of all professionals are valued.

“I think it’s trying to ascribe value to each discipline group so that they feel that they can contribute.”

(Educator X27)

However, not all interactions with other professions are positive. Educators needed to be aware of risks associated with responding to such difficult interactions with people from different professions.

“I guess if you’re voicing frustrations about certain things then that’s going to impact and perhaps add to a stereotype.”

(Educator K7)

### 3.4. Giving and Receiving Respect to Other Professions

Valuing own and others’ contributions were at the core of consciously role modelling respect for others’ professions.

“I think the way we get [the students to learn to work with other professions] is by being positive role models about [interactions with] other disciplines as educators and being mindful of what we say in front of the students we’re teaching whether they’re students of our own discipline or students of other disciplines.”

(Educator N4)

Such conscious portrayals of respect involved being mindful of what is said to students and how it is said.

“By the facilitators asking each other what they would do, it shows respect and an acknowledgement that we’re all part of a jigsaw and we’re all there for the patient.”

(Educator X27)

Respect was also perceived to be woven through a range of actions.

“You’d hope that in the whole way we treat them [other professions] … that they [the students] would sense that we do value [other professions’] input, according to the patient’s needs.”

(Educator Z1)

The notion of reciprocity was often embedded in respecting other professions.

“I think if you respect others, then it’s not unreasonable to expect respect in return.”

(Educator X27)

### 3.5. Being Learner-centred for Students’ Collaborative Practice

*Being learner-centred for students’ collaborative practice* provided an important sense of common purpose.

“We’re all really committed to creating these learning opportunities for the students.”

(Educator R20)

Seeing the benefits for students provides an important impetus for educators to continue to work together.

“There’s that synergy between the academics. … They want to work together and all the discipline academics are always thinking about how they can work together and how they can pull that together because they can all see the considerable benefits for their students to work in an interdisciplinary fashion.”

(Educator A3)

Although these five key behaviours were core to educators’ interprofessional relationships, their interrelated development was individual in nature. Based on experiences across different times and spaces, relationships between educators were not isolated to their current contexts and educational roles, or confined to their current colleagues. Relationships in the present were informed from current and past experiences and simultaneously shaped the development of relationships in different educational contexts.

## 4. Discussion

In making explicit the nature of educators’ interprofessional relationships we highlight five key behaviours which, while developed sequentially, were also revisited iteratively in accordance with individual circumstances. These behaviours resonated strongly with the interprofessional rapport building capabilities educators sought to develop in students [19]. Educators’ experiences across co-located university and healthcare settings informed the development of their behaviours and interprofessional collaborative relationships and, in turn, their educational strategies, and vice versa. The importance of these interrelated behaviours adds to the complexity of helping students learn to work with other professions. Consequently, pharmacy educators, like other educators working to help students learn to work with other professions, have individual and collective responsibilities to develop and role model positive interpersonal relationships.

Of particular interest to pharmacists are educators’ potential negative interprofessional experiences arising from problematic discipline territory delineations. For example, in spite of successful collaborations between pharmacists and other health professions, such as nursing [28,29] and medicine [12], and recognition of pharmacists’ roles in interprofessional healthcare teams [30], the pharmacy-medicine boundary is at times disputed. This boundary has been brought into focus in relation to prescribing rights [31] and vaccination by pharmacists [32]. A recent media release from the Australian Medical Association claimed that “*pharmacies have no proven record that they are safe or appropriate locations for such a private and potentially risky clinical procedure as vaccination*” [33]. Conversely, pharmacists have argued that “*although the concerns that physicians raise may be well intended, there is an undeniable territory struggle*” and those who enter into the debate “*must learn to check their vested interests at the door*” [32]. The risk of such professional “*vested interests*” to the formation of interprofessional relationships between health professionals and between health professional educators needs to be explicitly acknowledged in collaborative practice and preparation for collaborative practice. There is scope for each profession to explore how their own “*vested interests*” can be dealt with in a manner that does not impact negatively on educators’ interprofessional collaborative relationships, and ultimately how students learn to work with one another.

It can be argued that the development of educators’ interprofessional collaborative relationships in our research were facilitated by our co-located setting and through educators having roles across academic and clinical settings. Nevertheless, while potentially limiting its transferability to other settings that have less developed relationships, this research provides directions for future exploration of the nature of interprofessional collaborative relationships in settings with more distinct boundaries. Further research could explore the following questions: To what extent are the five interrelated interpersonal behaviours relevant for educators, including those from pharmacy, in non-co-located settings? In relation to previous negative experiences between professions, how can these five interrelated interpersonal behaviours be facilitated? How can pharmacy, as a profession, play a leadership role in helping pharmacy students in academic and clinical settings learn to work with other professions?

## 5. Conclusions

As an integral member of health profession collaborations, pharmacy educators are well placed to help students learn to work with other professions. However, educators are not immune from the challenges of interprofessional collaborative practice commonly experienced by clinicians. In embracing the complexity of IPE for WIL, educators need to ensure that the value of different professions’ contributions is maximized without previous negative collaborative experiences or vested professional self-interests being, as Coleridge so eloquently coined, the “albatross around one’s neck” [34].

## Figures and Tables

**Table 1:** Data sources.

Professional Category ^1^	Photo-Elicitation Interviews ^2^	Educators Contributing to Focus Groups ^3^
Diagnostic radiography	2	2
Medicine	6	3
Nursing	3	2
Nutrition and dietetics	4	3
Occupational therapy	2	2
Pharmacy	2	2
Physiotherapy	3	2
Speech pathology	2	1
TOTAL	24	19

Notes: ^1^ Professional categories are in alphabetical order; ^2^ Educators from academic (UONDRH) and clinical settings participated in photo-elicitation interviews in the initial study (reanalysed in current study). Male = 5, Female = 19. Photo-elicitation interviews in the initial study facilitated deep engagement with the topic of how students learn to work with other professions. Participants were asked to bring to the interview 10 photos of spaces, places or things they believed represented factors that influence how students learn to work with other professions. Interviews began with the request “*Can you take me through your photos please*” and ended with “*Is there anything else you would like to say?*” The interviewer’s prompt questions enabled interviewees’ perceptions and experiences to be explored in more depth. Questions and dialogue within the interview enabled the interviewer’s unfolding understanding to be made explicit and discussed with the participant. The number of interviews undertaken was sufficient to provide scope for a rich question and answer dialogue thus enabling deep interpretation of the phenomenon being explored; ^3^ Depending on their availability, educators participated in between one to four focus groups.
